# Cohort profile: MAVIE a web-based prospective cohort study of home, leisure, and sports injuries in France

**DOI:** 10.1371/journal.pone.0248162

**Published:** 2021-03-11

**Authors:** Madelyn Yiseth Rojas Castro, Ludivine Orriols, Benjamin Contrand, Marion Dupuy, Catherine Sztal-Kutas, Marta Avalos, Emmanuel Lagarde

**Affiliations:** 1 Bordeaux Population Health Research Center, University of Bordeaux, UMR U1219, INSERM, Bordeaux, France; 2 Calyxis, Centre of Risk Expertise, Niort, France; 3 SISTM Team Inria BSO, Talence, France; South China University of Technology, CHINA

## Abstract

MAVIE is a web-based prospective cohort study of Home, Leisure, and Sports Injuries with a longitudinal follow-up of French general population volunteers. MAVIE participants are voluntary members of French households, including overseas territories. Participation in the cohort involves answering individual and household questionnaires and relevant exposures and prospectively reporting injury events during the follow-up. Recruitment and data collection have been in progress since 2014. The number of participants as of the end of the year 2019 was 12,419 from 9,483 households. A total of 8,640 participants provided data during follow-up. Respondents to follow-up were composed of 763 children aged 0–14, 655 teenagers and young adults aged 15–29, 6,845 adults, and 377 people aged 75 or more. At the end of the year 2019, 1,698 participants had reported 2,483 injury events. Children, people aged 50 and more, people with poor self-perceived physical and mental health, people who engage in sports activities, and people with a history of injury during the year before recruitment were more likely to report new injuries. An interactive mobile/web application (MAVIE-Lab) was developed to help volunteers decide on personalized measures to prevent their risks of HLIs. The available data provides an opportunity to analyse multiple exposures at both the individual and household levels that may be associated with an increased risk of trauma. The ongoing analysis includes HLI incidence estimates, the determination of health-related risk factors, a specific study on the risk of home injury, another on sports injuries, and an analysis of the role of cognitive skills and mind wandering. Volunteers form a community that constitutes a population laboratory for preventative initiatives.

## Introduction

Home, Leisure, and Sports Injuries (HLIs) refer to all injuries occurring during private life apart from traffic injuries, occupational injuries, suicide, violence, or assault. These events are heterogeneous and elusive in their complexity and diversity. However, HLIs share recurring characteristics that suggest they can be prevented. HLI diversity is thus only apparent.

According to the European Injury Database (IDB), 232,000 people died from injuries in Europe (EU-28), between 2012 and 2014 [1]. HLIs caused around 49% of all trauma deaths. Falls accounted for 39%, poisoning for 13%, and burns for 4%. Among deaths related to falls, 72% involved people aged 65 years and more [1]. Children, older adults, and people with disabilities are population groups vulnerable to fatal and non-fatal HLIs [2, 3]. Burns and drowning are the leading causes of death among children [4]. Home injuries represent approximately one-third of the global burden of injuries [5, 6]. More than 21,000 deaths due to HLIs (3.8% of all deaths) occur annually in France. In 2012, the annual incidence rate in mainland France was estimated at 18% [7]. Hospitalizations for injuries incurred during physical and sports activities account for 18% of all hospitalizations due to HLIs [8].

The agenda for progress in preventing unintentional injuries [9] stresses the critical need for basic research to identify the burden of injuries, their causes and consequences in order to establish the evidence base necessary for effective intervention and prevention programmes. The extensive literature on risk factors for road traffic injuries and, to a lesser extent, occupational injuries [10], contrasts with a shortage of information on HLIs. This gap needs to be filled.

Multiple studies have identified health-related factors associated with falls in the elderly [11–13]. However, research on the impact of health risk factors among young and middle-aged adults is scarce [14]. Evidence of the effectiveness of home environmental modifications, including reducing falls at home among the elderly [15, 16], is still limited, mainly due to insufficient data [5, 6, 17].

Among children, HLIs are mainly due to drowning, falls, and fires [5]. These causes are known to be preventable by appropriate measures, e.g. improving parenting skills and a broader application of child restraint systems, pool fencing, smoke alarms, and window guards. The assessment of the impact of these interventions on the actual reduction of HLIs is very rarely conducted with adequate statistical power [18]. Among young adults, sports accidents are the subject of significant research. However, epidemiological studies are often conducted among high-level practitioners [19, 20]. Although the results of these studies also benefit casual practitioners, especially when establishing equipment standards and rules of the game, general population studies on the subject are scarce, especially for less common sports and leisure activities [21], while these studies are essential for making preventive actions more effective, targeting high-risk groups, and addressing high-risk products, behaviours and environments.

MAVIE is a multi-purpose web-based cohort established in 2014 for the surveillance and study of HLIs among the French general population. MAVIE was created in response to a lack of prospective information and detailed information on injury events in the EPAC French surveillance system [22], and in population surveys: *Santé et Protection Sociale* [23], *Décennale Santé* [24] and *Le Baromètre Santé* [25]. We needed prospective data, contextual information and relevant exposure data, including health condition, habits, lifestyle, and home environment. The project is conducted by a consortium created by a partnership between the Injury Epidemiology, Transport, Occupation (IETO) team of the Bordeaux Population Health Research Centre—INSERM U1219, and Calyxis, a non-profit association dedicated to risk prevention.

The objective of this article is to present the profile of the MAVIE cohort, outline the research questions, describe in detail the methodology used and the information available, the first results, plans and opportunities for collaboration.

## Cohort description

MAVIE is a web-based prospective cohort study with a longitudinal follow-up of HLIs.

The main objectives of the cohort are to determine the extent and characteristics of HLIs in France and to identify the factors associated with the occurrence and severity of HLIs. Achieving these objectives would allow us to propose priority intervention programmes and their evaluation among the cohort participants.

### Participants

MAVIE participants are members of French (including overseas territories) households of all ages who volunteered to join the cohort. The recruitment process began in November 2014 and is scheduled to stop at the end of 2020.

Cohort management was entirely online, including invitations, registration, and data collection. The largest share of participants was recruited through email invitations sent by three mutual insurance companies (MAAF, MACIF, and MAIF) to their insurees. A smaller proportion of the participants were informed of the MAVIE cohort and invited to participate through press releases, social media, posters, and flyers.

As a first step, we asked potential participants to choose a household reference member in charge of completing a web-based questionnaire for the household. In an attempt to address the foreseeable underrepresentation of the elderly who may have difficulties using computers, caregivers were invited to represent and participate on behalf of one older person. Only consenting members of each household were asked to provide individual information. Participation in the cohort involves answering questionnaires to provide information about individuals and their households and to report, prospectively, the HLI events during the follow-up period.

The inclusion criteria for the cohort were: 1) residing in France, 2) being able to answer the questionnaires in French, 3) having access to and being able to use the internet (at least the reference member).

### Follow-up

Events could be reported at any time on the website. Besides, every three months, the reference member receives a reminder email to invite him/her to report any injury events that may have happened to any household participant. If no event has occurred, a link in the email makes it possible to report with a single click, allowing us to confirm the follow-up. An invitation to report events was also included in the monthly cohort newsletter, and participants were invited to share their experience and opinion on a devoted forum. It was also possible to notify changes of residence and family composition. Finally, participants also have access to the beta version of the MAVIE-Lab, an app intended to provide personalized counselling to prevent HLIs [26].

### Data collection

Data are collected through self-reporting using a dedicated website (http://www.observatoire-mavie.com/) with password-protected private access. The questionnaire devoted to the household includes economic information, important family events, and habitat characteristics (interior and exterior), safety equipment, heaters, and electrical installations.

The individual questionnaire includes information on socioeconomic and demographic characteristics, typical daily schedule (time spent in each room of the house, time spent doing sport and leisure activities), sports, vacations, diseases in the past 12 months, medicines used during the month before inclusion, disabilities, health-related behaviours, mental and physical health perception and mental health indicators related to hyperactivity, mind wandering, depression (evaluated by the PHQ-9 Depression test questionnaire), anxiety (evaluated by the Generalized Anxiety Disorder 7-item scale), and drowsiness (evaluated by the Epworth Sleepiness Scale).

The injury events questionnaire included information on circumstances of the event (activity, place, time…), mechanisms of injury, health information in the two weeks before the injury (fatigue, anxiety, depression) and consequences (medical care, hospitalization, limitations…).

A questionnaire with specific information on sports (frequency, behaviours, equipment) was added after the recruitment. Similarly, an update of data related to health status, behaviours, and mental health indicators was requested during the March to May 2020 COVID-19 French lockdown in order to assess changes in mental health associated with lockdown conditions.

### Ethics

The French Data Protection Authority approved the protocol of this study. The study is declared to the CNIL under file number 912292. Identifying data were stored on servers located in a different location from those hosting the main database. Electronic informed consent was collected from all adult participants. Participation of children was done under the responsibility and with the consent of a legal guardian.

## Findings to date

### Recruitment and inclusion

**Fig 1:**
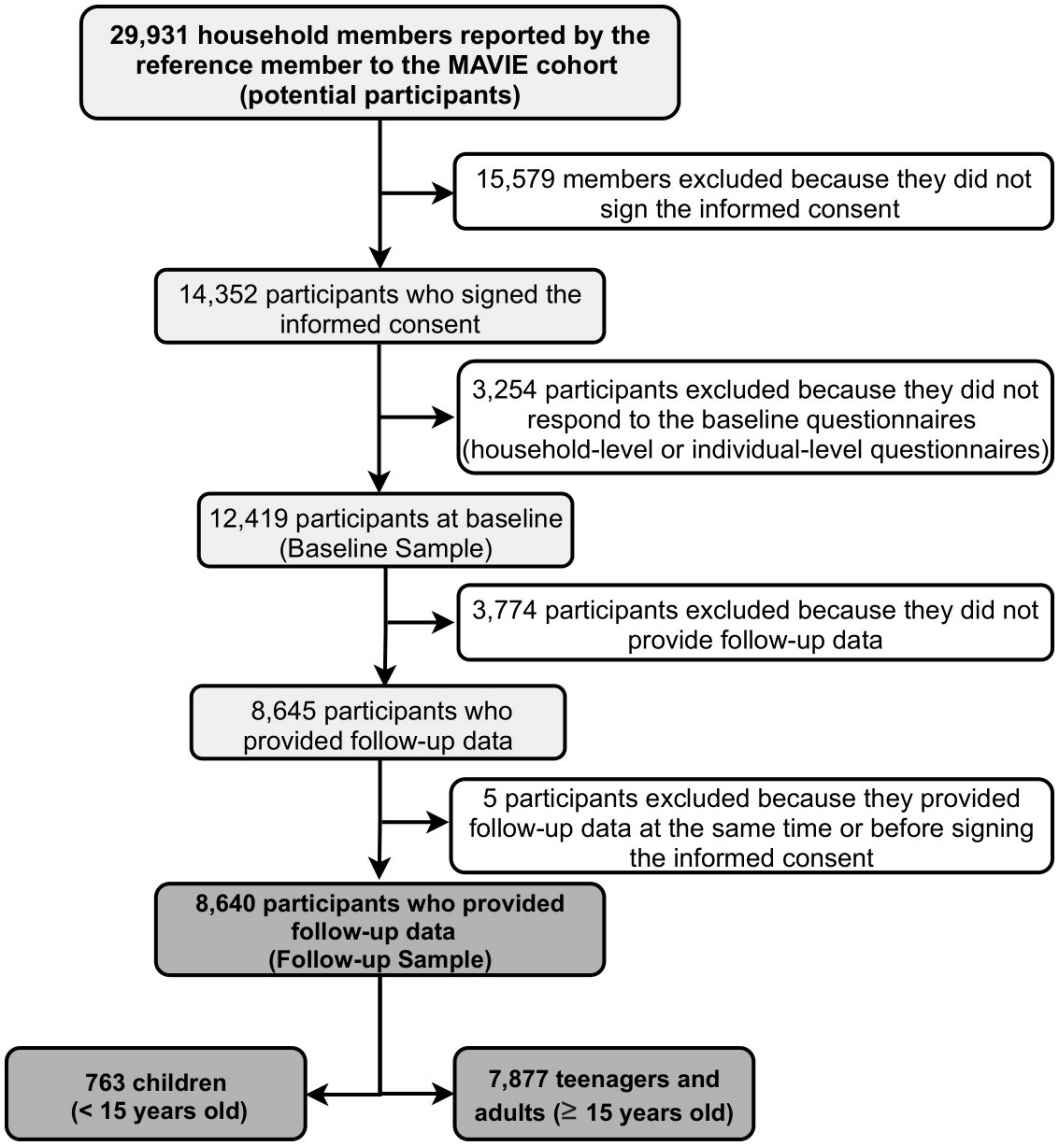
Flow diagram. Volunteer participation in the MAVIE cohort as of December 31, 2019.

Between November 2014 and June 2015, the mutual insurance companies sent 3,902,500 emails to their insurers, inviting household members to participate in the study. Between November 2014 and December 2019, around 30,000 eligible people were reported by a reference member. Among them, 14,352 people signed a consent form to participate in the study, and 12,419 people from 9,429 households responded to the baseline questionnaires. A total of 8,640 participants provided news at least once during the follow-up period (Fig 1).

During the follow-up, 317 participants left the cohort while allowing for the use of collected data. Twenty-nine of them died, as reported by the reference member, 22 of illness, one from suicide, one from an HLI, and five from unknown causes. The other 32 participants left the study due to changes in household composition. The remaining 256 participants did not report any reasons for leaving the study.

### Representativeness and baseline characteristics

Among the 12,419 participants who responded to the baseline questionnaires, 6,581 were female (53%) and 5,838 male (47%), their mean age was 49 and 53% of the participants were aged 50–74. MAVIE participants were older and had a higher socioeconomic and educational level than the French population (Table 1). Participants who achieved a diploma lower than a baccalaureate (BAC) were underrepresented in the sample (22% vs. 54% in France), and those who achieved a diploma BAC+3 or higher were overrepresented (61% vs. 28%). Only 14% of household reference members reported an annual income lower than the 30th percentile of French households.

**Table 1:** Comparison of the France population structure with the MAVIE baseline and follow-up sample structure.

	France Population in 2015		MAVIE baseline sample		MAVIE follow-up sample		
	*n*	(%)	*n*	(%)	*n*	(%)	P-value
All	66,422,469		12,419		8,640		
**Gender**							
Male	32,156,983	(48)	5,838	(47)	4,129	(48)	0.999
Female	34,265,486	(52)	6,581	(53)	4,511	(52)	
**Age**							
<15	12,339,441	(19)	999	(8)	763	(9)	**<0.001**
15–29	11,821,472	(18)	1,074	(9)	655	(8)	
30–49	17,257,411	(26)	3,251	(26)	2,018	(23)	
50–74	18,932,331	(28)	6,553	(53)	4,827	(56)	
≥ 75	6,071,814	(9)	542	(4)	377	(4)	
**Occupational Status (≥ 15 years old)**	54,042,754						
Farmer, operator, craftsman, shopkeeper	9,145,705	(17)	127	(1)	91	(1)	**<0.001**
Higher manager, professional occupations, independent	4,946,563	(9)	1,783	(18)	1,347	(19)	
Middle manager, employee	16,518,451	(30)	2,386	(25)	1,619	(23)	
Retired	14,543,422	(27)	3,819	(41)	3,017	(43)	
Unemployed	4,389,354	(8)	871	(9)	615	(8)	
Student	4,363,574	(8)	299	(3)	192	(2)	
*Other*	135,685	(0)	312	(3)	202	(3)	
*Missing data*			1,823	-	794	-	
**Highest education degree obtained**	48,165,255						
Less than General Baccalaureate	26,456,210	(55)	2,067	(22)	1,469	(21)	**<0.001**
General Baccalaureate or Diploma level BAC+2	8,085,172	(17)	1,569	(17)	1,111	(16)	
Diploma level BAC+3 or higher	13,623,873	(28)	5,665	(61)	4,276	(62)	
*Missing data*			3,118	-	1,784	-	
**Household size**	29,012,000		9,483		6,356		
1 member	10,299,260	(35)	2,198	(23)	1,563	(25)	0.470
2 members	9,544,948	(33)	4,211	(44)	2,953	(46)	
3 members	4,032,668	(14)	1,308	(14)	788	(12)	
4 members	3,423,416	(12)	1,222	(13)	756	(12)	
5 members	1,247,516	(4)	408	(4)	228	(4)	
6 members and more	464,192	(2)	113	(1)	53	(1)	
*Missing data*			23	-	15	-	
**Household income level** ^e^	29,993,595						
Low	8,609,667	(29)	1,177	(14)	704	(13)	**<0.001**
Middle	11,651,316	(39)	3,319	(41)	2,258	(41)	
High	9,732,612	(32)	3,651	(45)	2,598	(47)	
*Missing data*			1,336	-	796	-	

Sources from France population data in 2015: INSEE, demographic population estimates France 2015; INSEE, respondents to employment surveys (France excluding Mayotte, persons aged 15 or over); INSEE, respondents to census survey 2015; INSEE-DGFiP-Cnaf-Cnav-CCMSA, survey tax and social income 2015; Household income per decile of standard of living. P-values compare the cohort distributions (MAVIE follow-up sample) to the population distributions through the Chi2 test. In bold, P-values < 0.1.

The MAVIE participants aged 15 and over were more likely to report sport activities in the past 12 months (80%) than the French population of the same age (66%) [27]. In France in 2017, 10% of French people aged between 18 and 75 reported drinking alcohol every day, and 32% were smokers [28]. Among the MAVIE participants of the same age, 22% reported drinking alcohol four or more times a week, and 15% reported being smokers.

**Fig 2:**
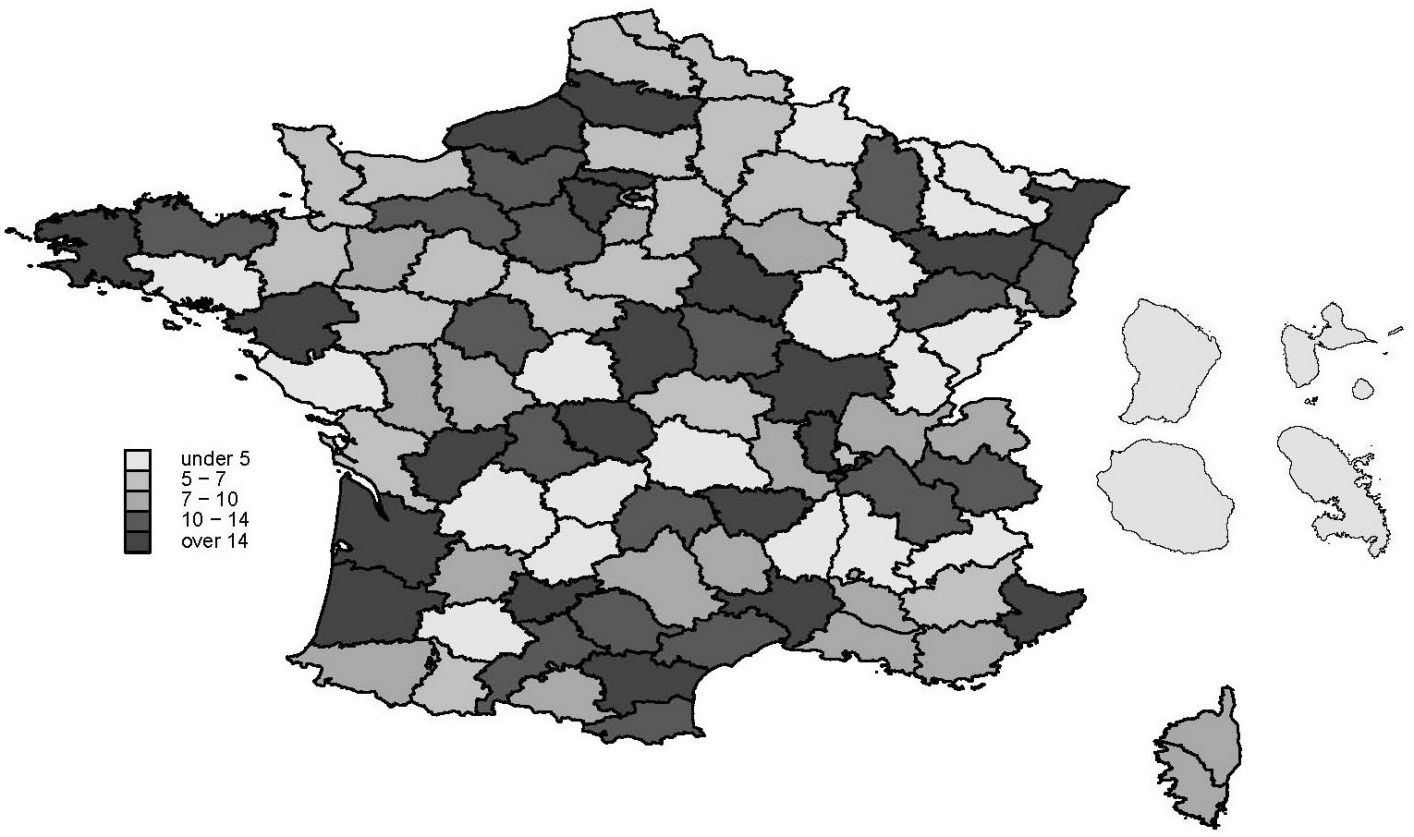
MAVIE cohort recruitment rate per 10,000 inhabitants in France and overseas territories according to departmental administrative areas as of December 31, 2019. Map data available under the open source license: Open Data Commons Open Database License (ODdL) by the OpenStreetMap Foundation ©OpenStreetMap contributors.

We recruited MAVIE participants over the whole of mainland France and French overseas territories, except Mayotte, where there are no offices of any of the insurance companies through which we made the recruitment. Participation in overseas territories was generally low. The participation rates at the departmental administrative area level ranged from 0.3 to 38.7 participants per 1,000 inhabitants (Fig 2).

Baseline socioeconomics and demographic characteristics of the baseline sample and of the follow-up sample were similar except that the youngest and oldest participants were less likely to be part of the follow-up sample (Table 1). When comparing the characteristics of follow-up respondents of all ages to those who voluntarily left the cohort, we found that the latter group were younger than those who stayed, with a lower education level and a lower perception of their physical health at the time of inclusion, suggesting that disease progression may be an important cause of dropout (Table 2).

**Table 2:** Comparison of the distribution of individual socioeconomic and demographics, characteristics, health behaviours, health status indicators, and previous home and leisure injuries among the follow-up sample and voluntary departures in the MAVIE cohort.

	MAVIE follow-up sample		Voluntary departures		
	*n*	(%)	*n*	(%)	P-value
All	8,640		304		
**Gender**					
Male	4,129	(48)	146	(48)	0.999
Female	4,511	(52)	158	(52)	
**Age (at baseline)**					
<15	763	(9)	18	(6)	**0.036**
15–29	655	(8)	53	(17)	
30–49	2,018	(23)	42	(14)	
50–74	4,827	(56)	155	(51)	
≥75	377	(4)	36	(12)	
**Occupational status (≥ 15 years old)**					
Farmer, operator, craftsman, shopkeeper	91	(1)	3	(1)	0.311
Higher manager, professional occupations, independent	1,347	(17)	41	(15)	
Middle manager, employee	1,619	(21)	41	(15)	
Retired	3,017	(38)	123	(46)	
Unemployed	615	(8)	22	(8)	
Student	192	(2)	26	(10)	
Other	202	(3)	13	(5)	
*Missing data*	794	-	14	-	
**Highest education degree obtained**					
Less than General Baccalaureate	1,469	(21)	92	(30)	**0.087**
General Baccalaureate or Diploma level BAC+2	1,111	(16)	35	(14)	
Diploma level BAC+3 or higher	4,276	(62)	132	(51)	
*Missing data*	1,784	-	45	-	
**Frequency of alcohol consumption (≥ 15 years old)**					
Less than 2 times a week	3,753	(58)	162	(67)	0.243
Two or more times a week	2,750	(42)	79	(33)	
*Missing data*		-	63	-	
**Tobacco (≥ 15 years old)**					
Smoker	843	(13)	24	(10)	0.563
Ex-smoker	1,399	(22)	42	(18)	
Non-smoker	4,240	(65)	174	(72)	
*Missing data*	1,340	-	27	-	
**Sport (last 12 months)**					
Yes	5,365	(81)	184	(74)	0.310
No	1,228	(19)	63	(26)	
*Missing data*	2,047	-	57	-	
**Self-perceived physical health at recruitment**					
Poor (1 to 4)	530	(8)	33	(14)	**0.020**
Good (5 to 7)	2,531	(38)	126	(52)	
Excellent (8 to 10)	3,545	(54)	85	(35)	
*Missing data*	2,034	-	60	-	
**Self-perceived mental health at recruitment**					
Poor (1 to 4)	422	(6)	22	(9)	0.690
Good (5 to 7)	2,150	(33)	72	(30)	
Excellent (8 to 10)	4,037	(61)	149	(61)	
*Missing data*	2,031	-	61	-	
**Injury history (during the last 12 months)**					
Yes	541	(9)	21	(10)	1.000
No	5,290	(91)	179	(90)	
*Missing data*	2,809	-	104	-	

^1^Level of education by age groups according to the census of the French population in 2015 (Low: < P50, High: ≥P50) (INSEE, 2015). Censure date to December of 2019. P-values compare the voluntary departure distributions to the cohort distributions (MAVIE follow-up sample) through the Chi2 test. In bold, P-values < 0.1.

During follow-up, 20% of the participants reported at least one HLI for a total of 2,483 reported HLIs. Table 3 compares injured and uninjured participants with respect to age, gender, self-perceived mental and physical health at recruitment, history of injury 12 months before inclusion, and sports practice. Children (<15 years old), people aged 50–74 and people aged 75 and more at the time of inclusion were more likely to report an HLI than adults aged 30–49. Adolescents and young adults aged 15–29 had the same risk as adults aged 30–49. People who practiced sport, people with poor self-perceived physical and mental health and those who reported at recruitment having had no HLI in the previous 12 months were also more likely to report an HLI during the follow-up.

**Table 3:** Comparison of the distribution of age, gender, self-perceived physical and mental health, previous home and leisure injuries and sports practice between injured and uninjured MAVIE participants.

	MAVIE participants who reported HLIs during the follow-up		MAVIE participants who did not report HLIs during the follow-up		
	*N*	(%)	*n*	(%)	OR (95% CI)
All	1,698		6,942		
**Gender**					
Male	806	(47)	3,323	(48)	Ref
Female	892	(53)	3,619	(52)	1.0 (0.9–1.1)
**Age (at baseline)**					
<15	184	(11)	579	(8)	**1.6 (1.2–2.0)**
15–29	119	(7)	536	(8)	1.1 (0.8–1.4)
30–49	353	(21)	1,665	(24)	Ref
50–74	953	(56)	3,874	(56)	**1.2 (1.0–1.4)**
≥75	89	(5)	288	(4)	**1.5 (1.1–2.1)**
**Sport (last 12 months)**					
Yes	1,309	(85)	4,467	(79)	**1.5 (1.3–1.8)**
No	240	(15)	1,163	(21)	Ref
*Missing data*	149	-	1,312	-	-
**Self-perceived physical health**					
Poor (1 to 4)	150	(9)	394	(7)	**1.5 (1.2–2.0)**
Good (5 to 7)	569	(37)	2,016	(36)	**1.1 (1.0–1.3)**
Excellent (8 to 10)	831	(54)	3,224	(57)	Ref
*Missing data*	148	-	1,308	-	-
**Self-perceived mental health**					
Poor (1 to 4)	112	(7)	324	(6)	**1.3 (1.0–1.8)**
Good (5 to 7)	484	(31)	1,747	(31)	1.0 (0.9–1.2)
Excellent (8 to 10)	955	(62)	3,564	(63)	Ref
*Missing data*	147	-	867	-	-
**Injury history (during the last 12 months)**					
Yes	200	(15)	425	(8)	**2.2 (1.7–2.8)**
No	1,116	(85)	4,611	(92)	Ref
*Missing data*	382	-	1,906	-	

Unadjusted ORs including a random effect to account for the cluster structure of households. **Abbreviations**: *n* = number of respondents, OR = odds ratio, CI = confidence interval. In bold, ORs with 95% CI excluding 1.

### MAVIE-Lab development

Data on exposure and risks were applied to develop an mHealth mobile application called MAVIE-Lab to assist in the personalized evaluation of HLI risk and to promote prevention measures. The first module of the app was developed for the risk of HLIs in sport. A beta version can be accessed online (https://ssl3.isped.u-bordeaux2.fr/MAVIE-OBS/Appli/#/).

The application comprises three modules:

1. A graphical overview comparing the average level of risk of different sports
2. An estimation of the personal injury risk for a selected sport
3. A graphical interactive overview showing the simulated impact on a selected number of preventative options [26].

### Ongoing activities and future plans

Four studies using data collected by the cohort are currently underway:

- HLI incidence estimation and characteristics
- Health-related and home-related risk factors
- Risk factors for sport injuries
- Cognitive skills, mind wandering and HLIs

Note that data on mental health collected in the cohort were leveraged to measure the impact of the COVID-19 national lockdown in March-May 2020 in France.

In the next stage of the cohort, we plan to simplify the questionnaires taking into account the results of the first stage and to move away from a household cohort approach to focus on individuals. We seek to boost the cohort with new recruitment through insurance companies but also by inviting volunteers from other large French cohorts. We are open to collaborations and proposals for new research questions by contacting the corresponding author or Calyxis.

## Strengths and weaknesses

MAVIE shares its main weaknesses with other web-based and voluntary-based cohorts: low response rate, volunteer bias, loss to follow-up and self-administered questionnaires. Table 4 summarizes each bias risk or limitation and the strategies we followed to reduce their effect.

**Table 4:** Summary of the risks of bias in the MAVIE cohort and strategies for its minimization.

	Bias risk or limitation	Strategies
**Sample size and participation bias**	Low response rate	Diversifying recruitment forms: mailing from mutual insurance, posters, media, and social networks Analysis conducted excluding injuries reported on the consent date Using a reference person and in particular caregivers for the elderly Incidence rates standardized according to sociodemographic characteristics
	Lack of representativeness	
	Particular interest in injury prevention or intellectual motivation	
	Risk of exclusion of non-users or infrequent users of the internet	
	Participation due to recent HLI for oneself or for a relative	
**Dropout**	Forgetting or losing interest in the study	Diversifying retention strategies: quarterly reminders, e-mails, monthly newsletter, online forum, smartphone application Using a reference person and in particular caregivers for the elderly
**Response**	Events under-reporting	Quarterly email reminders One-click event reporting. Reporting possible from the smartphone application
	Data are self-reported	Confidentiality and anonymity are guaranteed by the study investigators Events are collected prospectively
	Reliability of responses	Measuring exposures before the events report (prospective approach) Involving reference persons to communicate data on the elderly Assessing consistency of information
	Missing data	Questionnaires can be completed in several goes Imputation in data analysis

The response rate was very low. With almost 4 million emails sent, the response rate was only around 2 per 1,000. Further, investigating the motivations for participation in the cohort was not feasible because we cannot measure the demographic profiles of individuals exposed to the recruitment campaign. People who participate in health-related studies are often motivated by the benefits they might receive in terms of treatment or information [29, 30]. This let us assume that the volunteers were either intellectually curious or specifically interested in the topic of injury prevention, in particular if they had experienced a memorable injury. Participation prompted by a recent HLI could also have introduced a significant selection bias which may lead to misinterpreted associations between baseline health conditions and the subsequent risk of an HLI event.

Another possible motivation for participation that should be considered when assessing potential selection biases would be the positive feelings or better self-image that the act of participation may generate and the sense of loyalty or belonging associated with being part of a health study [29, 30].

Regarding the effect of internet use, we assumed at the design stage of the cohort that the level of internet access of the French population was sufficiently high (71% in 2010) and that the user profiles were sufficiently varied to obtain a representative sample. However, while internet accessibility may still be an obstacle to representativeness [30], we still think that the lack of motivation to participate had a much more important impact on representativeness and that few differences in the causes of HLIs between internet users and non-users can be expected.

Finally, another limitation common to other cohorts is the use of self-administered questionnaires, which can lead to selective responses and unreliable data [31]. Appropriate methods for the imputation of missing data are being considered in the analysis.

Despite these limitations, the web approach had its advantages in terms of costs and anonymity (Table 3). Web management of the cohort brought great flexibility to recruitment, cohort registration, data collection, and participant follow-up, overcoming several logistical and financial constraints [31]. We estimated that the average recruitment cost per participant was 10€, which is lower than the cost reported by offline recruitment methods [32].

Internet-based tools allowed us to reduce dropout by implementing different attraction and retention strategies as social networks and an online forum to share experiences and opinions, email reminders and a monthly newsletter. The web questionnaires allowed us to obtain detailed exposure and contextual information to study numerous risk factors and control by confounding factors. The website also facilitated updating and reporting changes in residence and in family composition.

The prospective nature of the cohort reduced recall bias [33, 34] and helped to apprehend potential causal pathways between exposure an events. Another strength is the availability of quantitative measures of a large range of exposures (e.g., time spent on sports, household activities, crafts and gardening, among others).

In summary, the self-selection of a sample of motivated participants certainly helped us get a better response to follow-up [35]. However, recruiting volunteers compromised the representativeness of the sample, with consequences for the generalizability of the results [36]. This will lead us to generalize with caution standardized incidence estimates to the general population. All associations observed between exposure and risk of HLI in this population will also be interpreted with this selection bias in mind. Stratification, weighting, and missing data management can help us make future results more transportable [36].
